# The London's Experiment with Paid Doctors

**Published:** 1919-03-08

**Authors:** 


### THE LONDON'S EXPERIMENT WITH PAID DOCTORS.

### Miss Luckes' Successor.

At the Quarterly Court of the Governors of the London Hospital the death of Miss Luckes, the late matron, was alluded to in sympathetic terms. The Governors were told that Miss Luckes was elected matron in 1880, and from that time has never ceased to work, literally night and day, for the uplifting of the nursing profession in general, and for the nursing in her hospital in particular. A number of copies of The Hospital were provided, for the use of the Governors, containing a full account of Miss Luckes' career.

Very careful consideration had been given to the appointment of a successor to Miss Luckes. It was felt that the appointment should be filled by a "Londoner," and choice fell upon Miss Monk, who had been at the hospital for fifteen years and was matron's chief assistant for 7½ years. During the period of Miss Luckes' long illness Miss Monk filled the post of acting matron with great success. When a vacancy occurred in the steward's department a year ago Miss Monk was elected steward by the Governors. This appointment she also filled to the complete satisfaction of the Committee. Her experience in the steward's department will be of the greatest possible use to her in the post of matron, especially with regard to the catering for the nursing staff. Miss Monk was a great personal friend and admirer of Miss Luckes, and will do all in her power to carry out the great traditions laid down by that lady.

### Military Beds.

The military wards were closed finally on January 15. During the war there have been treated 4,049 wounded men and 2,484 wounded officers at the hospital.

### The Cure of Syphilis.

Recently a most interesting demonstration took place in the syphilis department. The demonstration was intended to show that by means of the new treatment given for syphilis, 150 mothers, who during pregnancy were syphilitic, had not only been cured themselves, but had given birth to 150 perfectly healthy and normal babies. The demonstration was attended by many Government officials. One of the saddest parts of this work is the large number of perfectly innocent people who have contracted the disease. It is not too much to hope that with such work as the hospitals are now doing, this scourge will eventually be stamped out.

The Local Government Board and the London County Council have renewed their agreement with the London Hospital for the treatment of venereal disease for the year 1919, and are making a grant of £4,000 for this purpose.

### Resignation of Dr. Henry Head, F.R.S.

The Committee with regret report the resignation of Dr. Head, F.R.S., from active work at the hospital. Dr. Head had generously given up all private practice for the last three years and devoted the whole of his time to the work of this and other hospitals.

### Treatment of Pensioners.

The Committee has not thought it advisable to renew the agreement with the Ministry of Pensions for the treatment of pensioners discharged from the Army. The Committee feel that the treatment of pensioners is a duty for which the whole country is responsible, and adequate arrangements should be made for the treatment of pensioners, not as a favour by a charity, but as their undoubted right. They should be treated by the most skilled staff obtainable, who should be paid for their services, and arrangements ought to be made by which pensioners can get prompt and immediate treatment for their ailments, without the delay caused by the various formalities which have to be gone through at present. The Committee need hardly say that any pensioner coming to the hospital in urgent need of assistance will receive it promptly and efficiently like any other member of the public.

### The Future Medical Service of the Hospital.

The Committee reported on a very important matter which has been engaging their earnest consideration for a long time. Its importance lies in the fact that it touches a principle which has governed the work of voluntary hospitals in their treatment of the sick poor ever since voluntary hospitals have existed. They refer to the system by which the patients of the hospital are under the care of an honorary visiting staff.

This system, as applied to the London Hospital, is as follows:—

The beds of the hospital are divided into groups, and these groups are in the care of a physician and his assistant physician, or of a surgeon and his assistant surgeon. The physician and assistant physician, and also the surgeon and assistant surgeon, are known in hospital language as a "firm." Each "firm" has a share of men's, women's, and children's beds.

The "firms" visit the hospital officially usually on three days a week each, although many more visits may be made, and often are made, to see cases of urgency and sudden emergency. Attached to each firm is a house physician or house surgeon, who is resident in the hospital, and who watches over the cases of his chiefs between their visits. The Committee, now as always, acknowledge with gratitude and pride the magnificent way in which the honorary staff have hastened to the help of the charity in time of need. The staff have always upheld the dignity of their splendid profession, and have spread the reputation of this hospital far and wide.

### The System, Not Persons, Reviewed.

It is the devotion to duty of the medical and surgical staff, so freely given, that makes the task of the Committee the more difficult in suggesting any alteration in the system. They are most anxious that the Governors shall clearly distinguish between the system, which the Committee consider should be modified, and those who work in the system, the devoted servants of the hospital with a record of self-sacrifice which the public have almost come to take as a matter of course.

If medicine and surgery stood to-day in the position they stood fifty years ago, no improvement could be suggested in the system. Every patient was seen once or twice a week by a man of great experience, and his instructions were carried out by an officer resident in the hospital. This was an ideal arrangement under the conditions then prevailing.

But it is common knowledge that in the last twenty-five years discoveries of the greatest importance have been made in medicine and surgery, and these discoveries have entirely revolutionised treatment. For instance, the discovery of $x$-rays and their application to surgery in radiographic work and to medicine in treatment of various diseases; the advance of knowledge in bacteriology, and the application of this knowledge to the treatment and prevention of disease by vaccines and anti-toxins; the growth of information in the chemical processes taking place in the body—the chemistry of digestion for instance; investigations into the constitution of blood and of the blood pressure in the arteries; knowledge of the functions of nerves in relation to pain and to the mind, and how these functions are affected by disease—these and many others may be given as instances of the advancement in scientific knowledge. The cure of, and the prevention of, disease have become subjects for advanced technical knowledge and of continuous study. The patient is not to be regarded simply as a man or woman wanting to get well. The patient is worked at and his recovery fought for, and the experiences of the staff of half-a-dozen departments may be focussed upon his treatment.

### Prevention of Disease now a Chief Duty.

And closely connected with this scientific advance in the treatment of disease is the growing importance of the knowledge of the prevention of disease, perhaps one of the chief duties of a great hospital, and on which the future health of the nation depends.

And there is yet one other important matter to which a hospital such as the London must give its attention, and that is the education of the medical student, the man who is to be the doctor of the future. He must be trained in accurate observation and in the accurate recording of his observations in all these new departments.

With these great facts in mind, and these objects in view, the Committee and the medical staff have come to the conclusion that there must be some modification in the system of Medical Service, and they feel that this experiment may be made now that two vacancies happen to occur in the honorary medical staff by the retirement of Dr. Head and Dr. F. J. Smith. The Committee need hardly say that there is no question of changing the present honorary staff for a paid and whole-time staff. But the experiment may be made now that these vacancies occur. And the change would be this, that instead of appointing new members to the honorary staff, *whole-time* and adequately paid officers should be appointed to the beds which have become vacant, for it is realised that it is impossible for physicians and surgeons, who are in increasingly busy practice, to give so much time as is now required by their hospital duties.

### Experimental Modification of System.

Under the new arrangement the "firm" would consist of a director, three clinical assistants, laboratory assistance

**The London's Experiment with paid Doctors** (*cont.*).

and clerical assistance. All of these would be engaged for the whole of their time and would be paid for their services.

It is to be understood that this is not simply a teaching unit. It is a unit which will have to carry out precisely the same work as other parts of the hospital, admitting cases as they happen to come. The members of the staff of this unit would give their whole time to curing disease, research on the causes of disease, and the education of the medical students.

There are now two vacancies on the medical staff at the present moment, and there is the pending resignation of Mr. Jonathan Hutchinson on the surgical side. The Committee ask the permission of the Governors to make the experiment of filling these vacancies by the appointment of such a team of whole-time workers as has been described. If this scheme proves to be a sound and advantageous one, it may be repeated as vacancies occur.

Possible Future Reorganisation.

It may also be that whole-time units of very special departments, such as dermatological, syphilological, gynæcological, orthopædic and genito-urinary, may be formed on somewhat similar lines.

One thing the Committee wish to emphasise is that there will be no change whatever in the charitable work of the hospital, which is primarily to make sick people well. Neither will there be any change in its management, nor any interference by the Government.

One other point must be emphasised. This whole-time staff is in no sense put over the honorary staff, nor under the honorary staff. They must all remain, as the staff now is, colleagues of precisely equal rank, but serving the hospital in different ways, and able to do work of equal value.

The Committee do not wish to tie themselves down by saying that eventually the whole hospital shall be staffed by whole-time teams of paid officers, but permission is asked to make a limited experiment now. And the experiment will be tested by the results.

